# Dataset of prokaryotic and eukaryotic community structures from sediment and surface water eDNA of a sustainable mangrove fisheries (SMF) aquaculture pond

**DOI:** 10.1016/j.dib.2026.112800

**Published:** 2026-04-23

**Authors:** Punyasloke Bhadury, Anamitra Anurag Danda, Anwesha Ghosh, Yash, Nirupama Saini, Debmalya Roy Chowdhury, Soma Saha, Sourabh Kumar Dubey

**Affiliations:** a Integrative Taxonomy and Microbial Ecology Research Group, Department of Biological Sciences, Indian Institute of Science Education and Research Kolkata, Mohanpur, 741246, Nadia, West Bengal, India; b WWF-India, 1641 Madurdaha, Kolkata 700107, West Bengal, India; c WorldFish, Bhubaneswar 751025, Odisha, India

**Keywords:** Sustainable mangrove fisheries, Biological communities, eDNA, ONT sequencing

## Abstract

Sustainable mangrove fisheries (SMF) is an integrated mangrove fisheries approach used in coastal aquaculture ponds that brings sustainability to coastal aquaculture practices. To track the positive value of mangrove on the aquaculture practices, sediment and overlying surface water based environmental DNA (eDNA) biomonitoring has been initiated in SMF pond of Haroa Block (West Bengal, India), in close proximity to Sundarbans mangrove. In the post-monsoon of 2025 (January), sediment and overlying surface water samples were collected from the same SMF pond, followed by application of eDNA extraction and Oxford Nanopore Technologies (ONT) sequencing to deduce biological communities. During sampling, *in-situ* environmental parameters were recorded and dissolved nutrients were also measured. The dissolved nitrate and reactive silicate concentrations (320.32 and 46.85 µM respectively) were high in SMF pond reflecting effective mimicry of natural mangroves within the studied SMF pond. Using MinION platform integrating ONT, prokaryotic communities based on 16S rRNA metabarcoding revealed high abundance of Proteobacteria, followed by Firmicutes which were higher in sediment compared to surface water. There was high abundance of Bacteroidetes and Actinobacteria in sediment compared to the surface water reflecting the potential pool of complex forms of organic carbon. Microphytobenthos in the sediment was represented by members of Cyanobacteria. At the 18S rRNA level, members of Bacillariophyta dominated both sediment and water reflecting their importance in photosynthetic primary production and potential food for shrimps growing in the SMF pond. Besides, sequences representing other groups of alveolates were also encountered in sediment and surface water. The presence of Ascomycota and Basidiomycota within the eukaryotic pool reflecting their specific role in breakdown and utilization of mangrove litter, in addition to Firmicutes. This study generates key baseline information for long-term monitoring and represents the first eDNA-based dataset for sediment and surface water of prokaryotic and eukaryotic biological communities within the SMF.

Specifications Table

**Table:** 

Subject	Earth & Environmental Sciences
Specific subject area	Environmental science
Type of data	Raw and analyzed
Data collection	During sampling, *in-situ* environmental parameters were measured in triplicate using handheld digital probes with ATC configuration. Surface water samples (1 L) were collected in wide-mouth sterile amber bottles and fixed with buffered 4% formalin for dissolved nutrient estimations. Sediment samples (100 mL) were collected using a handheld corer (10 cm length, 3.5 cm diameter), and 1 L surface water samples were subsequently collected from the SMF pond in wide-mouth amber bottles. Both sediment and water samples were fixed with absolute molecular-grade ethanol for characterization of microbial community signatures using environmental DNA (eDNA) metabarcoding approaches. For the estimation of dissolved organic carbon (DOC), 40 mL of filtered and unfixed water was collected in glass vials with open-top, pierceable caps enabled with butyl rubber septa. Environmental DNA (eDNA) was extracted from collected sediment and surface water samples to examine prokaryotic and eukaryotic communities.
Data source location	Region: Haroa Block, Basirhat Subdivision, North 24 Parganas, West Bengal Country: India Latitude and Longitude: 22°36′16.8″N 88°44′06.4″E
Data accessibility	Repository name: Bioproject number PRJNA1300388 submitted to NCBI Data identification number: Direct URL to data: https://www.ncbi.nlm.nih.gov/bioproject/?term=PRJNA1300388

## 1 Value of the Data

• The dataset on prokaryotic and eukaryotic communities representing both the sediment and surface water is the first sequence-based baseline generated globally from a sustainable mangrove fisheries pond.
• The generated dataset also represents multiple environmental parameters (*in-situ* physical, chemical and biological) and thus indicating the importance of SMF pond in mimicking natural conditions prevalent within mangroves that can lead to sustainable shrimp aquaculture practices.
• The generated dataset can act as a biological proxy for assessing positive impacts of mangroves on the biodiversity within a sustainable mangrove fisheries pond and linked blue economy.
• The generated datasets also deepen our understanding of the functional interplay between prokaryotic and eukaryotic communities in sediment and surface water within a SMF pond.
• The study provides a snapshot of the SMF approach integrating biological communities that can be adopted to restore abandoned coastal aquaculture ponds.
• The dataset will be crucial for scientific community and ecosystem managers for developing targeted restoration of abandoned coastal aquaculture ponds which were created by clearing natural mangroves and will help achieve goals of carbon sequestration.

## 2 Background

Aquaculture production is projected to surpass wild-capture fisheries and will be the primary source of aquatic animal protein for global population in order to meet SDG targets. Farmed shrimp grown in shrimp aquaculture ponds across Asia and also parts of South America have been widely criticized for causing mangrove loss. There are calls globally to implement more sustainable aquaculture approaches that protect mangroves. In the last few decades, 26.7% of the global mangroves have been lost due to unsustainable coastal aquaculture practices [1]. This has resulted in extensive transformations of landscape across coastlines globally [2]. Sustainable mangrove fisheries (SMF) is an integrated mangrove fisheries approach used in aquaculture ponds that brings sustainability to coastal aquaculture practices. Over the last century, Sundarbans, world’s largest contiguous mangrove wetland which is a Ramsar site and an UNESCO World Heritage Site, has experienced loss of mangrove cover [3]. This is due to the destructive practice of conversion of mangroves which are not part of the protected areas of Sundarbans, into coastal shrimp aquaculture ponds. Besides, to enhance production, use of feeds and antibiotics can affect surrounding aquatic biodiversity along with wildlife. Sustainable Mangrove Fisheries (SMF), utilizes mangrove vegetation including the beneficial importance of mangrove litter to promote sustainable aquaculture, restore ecosystem services, and improve livelihoods of shrimp aquaculture farmers as well as contribute to overall biodiversity. Detailed profiling of biological communities from sediment and surface water eDNA metabarcoding reflecting the benthic to pelagic layers could be crucial that can enhance our understanding of the positive consequences of SMF towards achieving sustainable shrimp farming practices. This study provides a glimpse of the structure of prokaryotic as well as eukaryotic communities in a SMF pond reflecting the sediment and overlying water (surface water) along with generation of baseline information on key environmental parameters.

## 3 Data Description

The dataset encompasses *in-situ* environmental parameters, along with concentrations of estimated dissolved nutrients and dissolved organic carbon (DOC) from surface water of the studied SMF pond (Table 1). The dissolved nutrients, particularly nitrate and reactive silicate concentrations were higher in SMF pond that could support primary production along with influencing stoichiometrically favoured nutrient cycling. Besides, measured pH and TDS values in SMF reflected the mimicking of environmental conditions generally found in estuarine mangroves.

**Table 1:** Environmental parameters recorded from the surface water of SMF pond located in Haroa, West Bengal, India.

Environmental parameters	SMF Pond
**Ambient temperature (AT) (°C)**	25.37 ± 0.06
**Surface water temperature (SWT) (°C)**	17.80 ± 0.01
**Dissolved Oxygen (DO) (mg/L)**	3.06 ± 0.11
**pH**	6.703 ± 0.00
**Total Dissolved Solids (TDS**)**(ppm)**	1390.67 ± 3.03
**Electrical Conductivity (EC) (µS/cm)**	2764.67 ± 5.03
**Salinity**	1.35 ± 0.00
**Secchi Depth (cm)**	33 ± 0.00
**Dissolved ammonia (µM)**	0.52 ± 0.00
**Dissolved nitrate (µM)**	320.32 ± 0.00
**Dissolved ortho-phosphate (µM)**	46.85 ± 0.00
**Reactive** s**ilicate (µM)**	32.57 ± 0.00
**Dissolved Organic Carbon (DOC) (mg/L)**	122.50

This dataset also explains the observed structure of prokaryotic and eukaryotic communities derived from sediment and surface water eDNA collected from the same SMF pond deduced using 16S rRNA and 18S rRNA metabarcoding. Approximately, 1 GB of raw data was generated that was cleaned up by removing adapters and barcodes; number of reads for sediment and surface water detailed in Table 2.

**Table 2:** Number of reads generated for sediment and surface water from SMF pond following QC.

Sample name	Sample type	Amplicon	Total reads
SMF Sediment	Sediment	16S rRNA	575,872
SMF Water	Surface Water	16S rRNA	98,223
SMF Sediment	Sediment	18S rRNA	48,825
SMF Water	Surface Water	18S rRNA	263,027

**Fig. 1:**
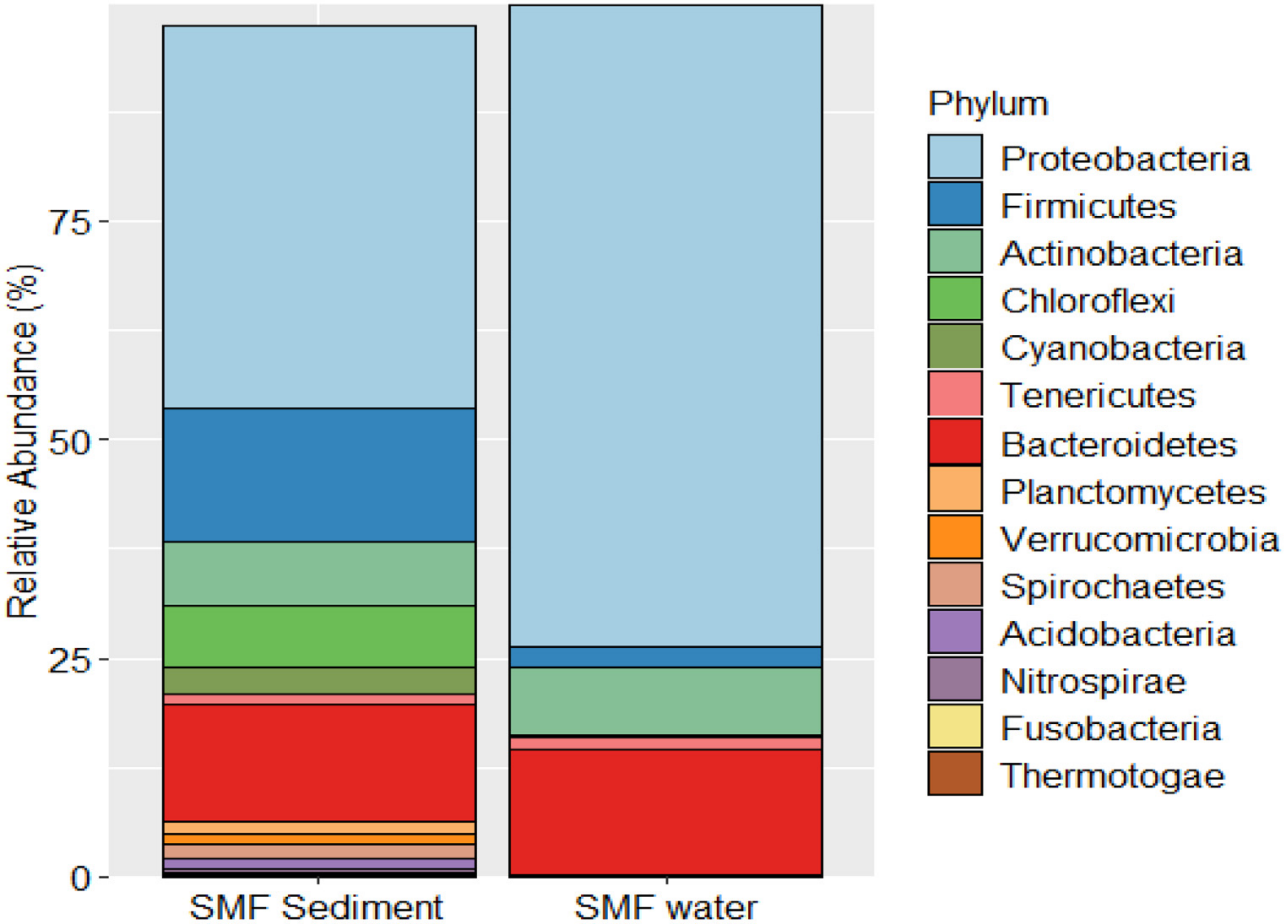
Prokaryotic communities deduced from sediment and surface water of SMF pond using 16S rRNA metabarcoding approach.

The observed prokaryotic communities in sediment and overlying water (surface water) based on 16S rRNA metabarcoding revealed distinct pattern within the studied SMF pond (Fig. 1). The bacterial communities were dominated by members of Proteobacteria both in sediment and overlying water (surface water) in the SMF pond. The high abundance of members of Firmicutes, particularly, in the sediment reflect the potential role of breakdown of nutrient pools including complex forms of carbon as well in the surface water due to deposition of mangrove litterfall. Hence, the observed DO level in surface water was found to be on the lower side. The DOC pool was high (Table 1) reflecting favourable conditions for members of Firmicutes to thrive across sediment to water columns and thereby driving productivity as observed in earlier studies [4,5]. Besides, abundance of Bacteroidetes and Actinobacteria were also high in sediment and water reflecting the availability of forms of complex organic carbon pool. The presence of members of Cyanobacteria in sediment reflected the potential presence and importance of microphytobenthos which can serve as a food for many groups including shrimps and other members of crustaceans within the studied SMF pond. The presence of members of *Nitrospira* and Planctomycetes in SMF sediment reflect the process of nitrogen cycling and potential synergy with carbon cycling that can maintain ecological health of SMF ponds. In general, both in sediment and surface water observed microbial communities mimicked the communities that are presentin mangroves such as Sundarbans.

**Fig. 2:**
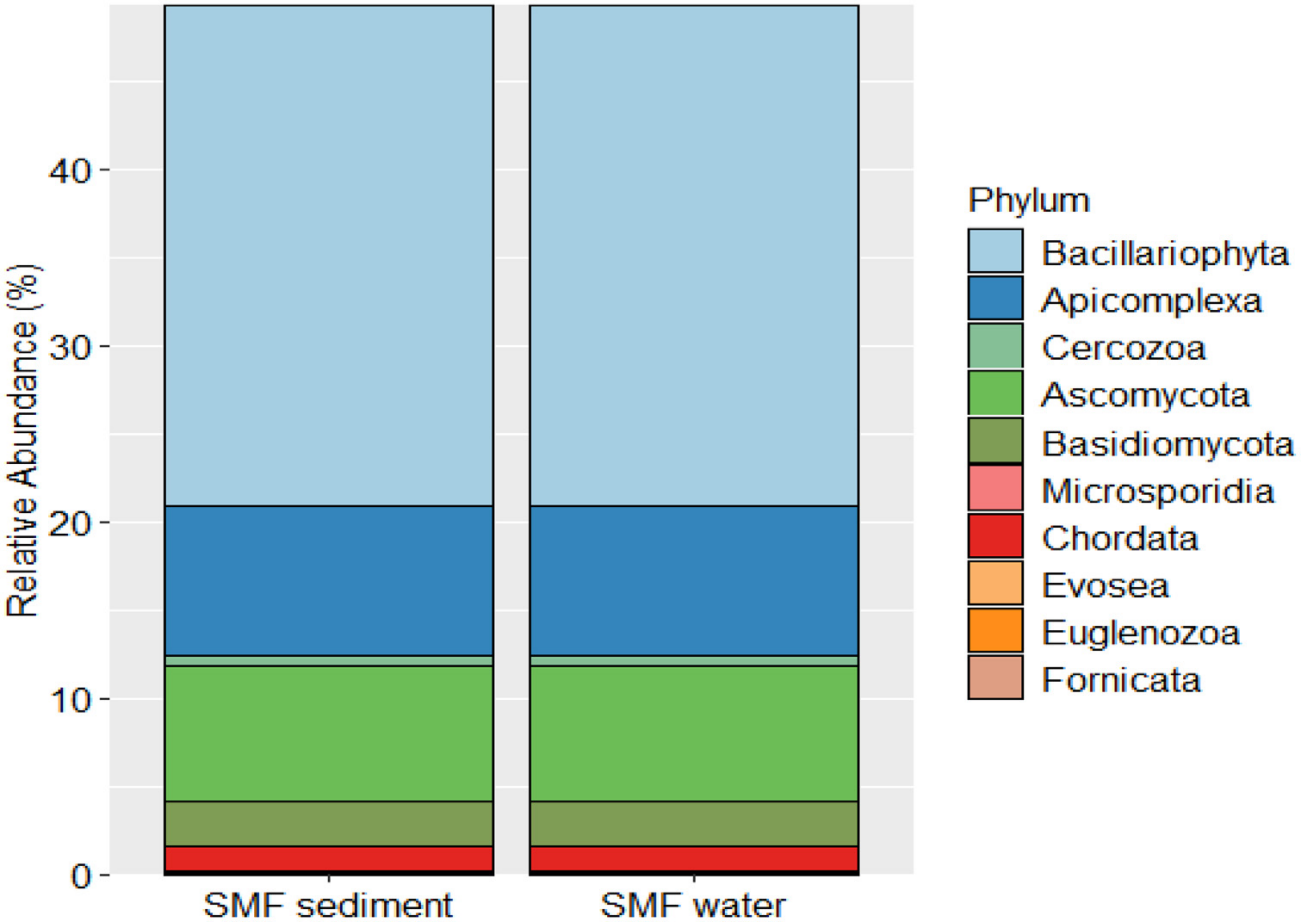
Eukaryotic communities deduced from sediment and surface water of SMF pond using 18S rRNA metabarcoding approach.

At the 18S rRNA level, distinct biological communities were observed in the sediment and overlying surface water (Fig. 2). There was very high abundance of members of Bacillariophyta (diatoms) both in sediment and overlying surface water (Fig. 2). The presence of diatoms in the sediment could be representative of the microphytobenthos. The prevalence of diatoms with such high abundance in the SMF pond reflects the mimicking of natural mangroves where diatom communities drive photosynthetic primary production. Besides, this group play an important role in trophic transfer including food for shrimps that are part of SMF aquaculture practices. Besides, alveolates such as Apicomplexa and Cercozoa were found in higher abundance in both sediment and surface water reflecting their contribution to the trophic transfer of energy. The presence of Ascomycota and Basidiomycota sequences both in sediment and water reflected their functional importance in breakdown of mangrove litter. The presence of Fornicata sequences reflected that other members that make up biodiversity pool in mangroves such as members of molluscs have also colonized the SMF pond reflecting the synergy and efficient mimicry of mangrove aquaculture-based shrimp farming. Permutational multivariate analysis of variance (PERMANOVA) based on Bray–Curtis revealed significant niche-specific differences in both bacterial and eukaryotic communities within the SMF ponds (*p* < 0.05). Bacterial communities exhibited broader phylum-level distributions, whereas eukaryotic communities were comparatively more conserved across sediment and water habitats. The overall dataset is available in NCBI under BioProject number PRJNA1300388.

## 4 Experimental Design, Materials and Methods

### 4.1 Sampling

In the Haroa Block under Basirhat Subdivision of North 24 Parganas (West Bengal, India), sampling was undertaken in a SMF pond of approximately five years into existence. The sediment and surface water were collected from the SMF pond in January, 2025. Approximately, 100 mL of sediment samples were collected using a handheld corer (corer dimensions: length-10 cm and diameter- 3.5 cm). Sediment samples were collected for undertaking sediment eDNA extraction and immediately fixed with molecular-grade absolute ethanol (Merck, Germany). Briefly, 1 L of surface water was collected and immediately fixed with molecular grade ethanol (Merck, Germany) following published methodologies. From the SMF pond, 1 L of surface water was separately collected and immediately fixed with buffered 4% formalin (Merck, Germany) to estimate dissolved nutrients (ammonia, nitrate, ortho-phosphate, and reactive silicate). Unfixed, filtered surface water samples were collected for dissolved organic carbon (DOC) immediately transported in ice-packed after collection for further analyses.

### 4.2 Measurement of *in-situ* environmental parameters

At the time of sampling, *in-situ* environmental parameters were measured for surface water of SMF pond. The air temperature (AT,°C) and surface water temperature (SWT,°C) were recorded using a digital thermometer (Digi-sense RTD meter 20,250–95, single-input thermometer with NIST-traceable calibration). Dissolved oxygen (DO, mg/L) was measured with a hand-held probe (Hanna Instruments HI98193, EU, with temperature sensor), while pH was determined using a pH probe (Hanna Instruments HI98190, EU, with temperature sensor). Salinity, total dissolved solids (TDS, ppm), and electrical conductivity (EC, µS/cm) were measured with a multiparameter probe (HI98192 EC/TDS/Resistivity/Salinity Meter, with temperature sensor, Hanna Instruments, EU). Water transparency (Secchi depth, cm) was estimated using a Secchi disc (LaMotte, France). All instruments were calibrated both in the laboratory and in the field, following the manufacturers’ protocols. Measurements were undertaken in triplicates during the day of sampling.

### 4.3 Estimation of dissolved nutrients in surface water

Surface water samples were filtered through 0.22 µm pore size, 25 mm diameter nitrocellulose syringe filters (Whatman Uniflo; United Kingdom). Dissolved nutrients, including ammonia, nitrate, ortho-phosphate and reactive silicate, were measured by UV–Vis spectrophotometer using standard colorimetric methods [6]. All analyses were conducted in triplicates.

### 4.4 Estimation of dissolved organic carbon (DOC) in surface water

Surface water samples were filtered through 0.22 µm pore size, 25 mm diameter nitrocellulose syringe filters (Whatman-uniflow, United Kingdom) and DOC was measured by thermos catalytic combustion at 850 °C using a multi N/C 2100S analyzer (Analytik Jena AG, Germany). All analyses were performed in triplicate, with the coefficient of variation (CV) maintained below 4% for each sample run [7,8].

### 4.5 Extraction of environmental DNA (eDNA) and Nanopor**e** sequencing

Sediment eDNA was extracted using HiPurATM Soil DNA purification kit (Himedia, India) following methodological modifications [9]. Surface water of 1 L was collected from two points and were fixed with molecular grade ethanol on site. The biomass from surface water was concentrated in 0.22 µm 47 mm nylon filter paper using a vacuum pump and the filter papers were stored at −20 °C until further processing. The eDNA from surface water was extracted using modified Bostrom et al. protocol [10,11]. The extracted sediment and surface water eDNA samples were run on 1% agarose gel. The DNA concentration was quantified using a Nanodrop 2000c Spectrophotometer (Thermo Scientific, United States of America) and Qubit 1X dsDNA HS (High sensitivity) Assay kit (Thermofisher Scientific, United States of America). The 18S and 16S rRNA regions were amplified using UniF (ACCTGGTTGATCCTG) and UniR (TGATCCTTCYGCAGG) as well as FC27 and 1384R primers respectively (GCC BIOTECH, India) from extracted eDNA. PCR conditions included initial denaturation at 95 °C for 5 min, 35 cycles of denaturation at 95 °C for 30 s, annealing at 58 °C for 30 s, and extension at 72 °C for 2 min, followed by a final extension at 72 °C for 5 min. Library preparation was performed using Oxford Nanopore Technologies (ONT) ligation sequencing chemistry, involving the SQK-LSK109 ligation sequencing kit (Oxford Nanopore Technologies, Oxford, United Kingdom) and EXP-PCR096 native barcoding kit (Oxford Nanopore Technologies, Oxford, United Kingdom). The sequencing was performed in the Nanopore MinION (Oxford Nanopore Technologies, Oxford, United Kingdom) using SpotON Flowcell R9.4 (FLO-MIN106). Base-calling and demultiplexing of raw reads in FASTQ format were carried out using Guppy v2.3.4 (available at https://community.nanoporetech.com).

### 4.6 Data analyses

The ONT-generated raw reads were concatenated using the ``Concatenate multiple datasets'' tool in the Galaxy platform (usegalaxy.eu)**.** Adapter trimming of the ONT reads were performed using Porechop [12], while quality control was conducted utilizing NanoPlot [13] and Filtlong [14]. Following QC, taxonomic classification of the reads was performed using available sequence databases including SILVA. Reads were mapped to reference sequences using BLASTn, applying an identity threshold of ≥90–96% for taxonomic assignment and an e-value cutoff of ≤1e-5 (0.00001). Permutational multivariate analysis of variance (PERMANOVA) based on Bray–Curtis was undertaken for both bacterial and eukaryotic communities within the SMF ponds.

## Limitations

Not applicable.

## Ethics Statement

The work described above did not involve human or animal subjects; therefore, no regulatory compliance guidelines were applicable.

## CRediT Author Statement

Punyasloke Bhadury, Anamitra Anurag Danda and Yash undertook field sampling; PB, AG, Yash and NS performed the experimental work and PB drafted the initial version of the manuscript. PB and AAD contributed to the conceptualization, validation of analysis and edited the manuscript. AG and Yash edited the manuscript. DRC, SS and SKD contributed to the field sampling and edited the manuscript.

## Declaration of Competing Interest

The authors declare that they have no known competing financial interests or personal relationships that could have appeared to influence the work reported in this paper.

## Data Availability

Profiling of biological communities in Sustainable Mangrove Fisheries (SMF) pond using eDNA approach (Original data).
